# Deletion of Smad3 protects against C-reactive protein-induced renal fibrosis and inflammation in obstructive nephropathy

**DOI:** 10.7150/ijbs.62929

**Published:** 2021-09-21

**Authors:** Yong-Ke You, Wei-Feng Wu, Xiao-Ru Huang, Hai-Di Li, Ye-Ping Ren, Jin-Cheng Zeng, Haiyong Chen, Hui Yao Lan

**Affiliations:** 1 Department of Nephrology, Shenzhen University General Hospital, Shenzhen University, Shenzhen, Guangdong, China.; 2 Department of Medicine and Therapeutics, and Li Ka Shing Institute of Health Sciences, the Chinese University of Hong Kong, Hong Kong, China.; 3 School of Chinese Medicine, Li Ka Shing Faculty of Medicine, the University of Hong Kong, Hong Kong, China.; 4 Guangdong-Hong Kong Joint Laboratory for Immunological and Genetic Kidney Disease, Guangdong Academy of Medical Science, Guangdong Provincial People's Hospital, Guangzhou, China.; 5 Dongguan Key Laboratory of Medical Bioactive Molecular Developmental and Translational Research, Guangdong Provincial Key Laboratory of Medical Molecular Diagnostics, Guangdong Medical University, Dongguan, Guangdong, China.; 6 CUHK-Guangdong Provincial People's Hospital Joint Research Laboratory for Immunological and Genetic Kidney Disease, the Chinese University of Hong Kong, Hong Kong, China.

**Keywords:** C-reactive protein, renal fibrosis and inflammation, TGF-β/Smad3, NF-κB, UUO

## Abstract

**Introduction and Aims:** Elevated plasma levels of C-reactive protein (CRP) are closely associated with progressive renal injury in patients with chronic kidney disease (CKD). Here, we tested a hypothesis that CRP may promote renal fibrosis and inflammation via a TGF-β/Smad3-dependent mechanism.

**Methods:** Role and mechanisms of TGF-β/Smad3 in CRP-induced renal fibrosis and inflammation were examined in a mouse model of unilateral ureteral obstruction (UUO) induced in CRP Tg/Smad3 KO mice and in a rat tubular epithelial cell line in which Smad3 gene is stably knocked down (S3KD-NRK52E).

**Results:** We found that mice overexpressing the human CRP gene were largely promoted renal inflammation and fibrosis as evidenced by increasing IL-1β, TNF-α, MCP-1 expression, F4/80^+^ macrophages infiltration, and marked accumulation of α-smooth muscle actin (α-SMA), collagen I and fibronectin in the UUO kidney, which were blunted when Smad3 gene was deleted in CRPtg-Smad3KO. Mechanistically, we found that the protection of renal inflammation and fibrosis in the UUO kidney of CRPtg-Smad3KO mice was associated with the inactivation of CD32-NF-κB and TGF-β/Smad3 signaling.

**Conclusion:** In conclusion, Smad3 deficiency protects against CRP-mediated renal inflammation and fibrosis in the UUO kidney by inactivating CD32-NF-κB and TGF-β/Smad3 signaling.

## Introduction

Renal fibrosis is the common pathway of all progressive chronic kidney diseases (CKD) that finally leads to end-stage renal disease (ESRD) 1-5. Increasing evidence shows that obstructive nephropathy leads to proximal tubular cell loss and interstitial fibrosis. The unilateral ureteral obstruction (UUO) model is widely used to study the mechanisms of tubulointerstitial fibrosis via surgically induced unilateral obstructive renal injury. UUO triggers a sequence of events in the obstructed kidney including interstitial inflammation, infiltration with pro-inflammatory cytokines such as interleukin-1 beta (IL-1β), interleukin-6 (IL-6), tumor necrosis factor-α (TNF-α), and monocyte chemoattractant protein-1 (MCP-1), as well as accumulation of fibrotic markers such as collagen I, fibronectin and α-smooth muscle actin, ultimately leading to progressive renal injury. Chronic inflammatory conditions in CKD patients strongly correlate with elevated levels of C-reactive protein (CRP); emerging evidence indicates that CRP acts as one of the most essential inflammatory markers and mediators in various chronic diseases such as CKD, diabetic kidney diseases, and cardiovascular diseases 6-12. However, the signaling mechanisms by which CRP exacerbates the progression of CKD remains to be elucidated.

We have previously shown that mice overexpressing the human CRP gene (CRP Tg) largely promotes ischemia/reperfusion-induced acute kidney injury (AKI) and UUO-induced renal inflammation and fibrosis 6, 13. These observations suggest that CRP is not only an inflammation biomarker, but also pathogenic for triggering renal inflammation and fibrosis. Further studies found that CRP-mediated renal inflammation and fibrosis in UUO kidneys in CRP Tg mice is associated with activation of NF-κB and TGF-β/Smad3 signaling 6. *In vitro* studies also showed that the addition of CRP activates NF-κB and TGF-β/Smad3 signaling to induce renal inflammation and fibrosis 8. However, the exact mode of CRP signaling in renal inflammation and fibrosis remains to be determined. Thus, the present study examined the potential importance of TGF-β/Smad3 signaling in a CRPtg mouse model of UUO by conventionally deleting Smad3.

## Materials and methods

### Animal model

Ten-week-old male human CRP Tg and Smad3 KO mice were used in this study. Phenotypes of both mouse strains have been described previously 13-15. CRP Tg/Smad3 KO mice and their littermates control mice (CRP Wt/Smad3 Wt, CRP Wt/Smad3 KO, CRP Tg/Smad3 Wt) were generated by the crossbreed of Smad3 heterozygous and CRP Tg mice as previously reported by us 13. Progressive kidney disease was induced in a mouse model of unilateral ureteral obstructive nephropathy (UUO) in groups of 6-8 mice by the left ureteral ligation as described previously 16, 17. All mice were sacrificed on day 7 after the left ureter ligation. Blood was collected for the detection of renal function. Kidney tissues were collected for histology, immunohistochemistry, western blotting, and real-time reverse transcript-PCR analysis. All experimental procedures were approved by the Animal Experimentation Ethics Committee of The Chinese University of Hong Kong, and experimental methods were performed by approved guidelines.

### Cell culture

NRK-52E rat renal tubular epithelial cells (TECs) were obtained from the American Type Culture Collection (Manassas, VA, USA) and were cultured in DMEM (Dulbecco's modified Eagle's medium)/Ham's F12 medium (Invitrogen Life Technologies, Gaithersburg, MD, USA) supplemented with 10% fetal bovine serum (Invitrogen Life Technologies, Gaithersburg, MD, USA) until sub-confluent. To investigate the role of Smad3 signalling in CRP-induced TEC fibrosis, a stable Smad3 gene knockdown NRK52E TEC line was used 17-19. Smad3 knockdown cells were cultured with or without CRP (10μg/ml) and the role played by Smad3 in CRP-induced fibrosis was determined. Each experiment was repeated independently at least three times.

### Renal function and morphological changes

Serum creatinine and blood urine nitrogen were determined by quantitative enzymatic methods (Stanbio Laboratory, Texas, USA). Renal morphology changes were examined in formalin-fixed, paraffin-embedded sections (3 µm) with Periodic Acid-Schiff's (PAS) reagent or a Masson's trichrome staining kit (ScyTek Laboratories, West Logan, UT) according to the manufacturer's instructions and as previously described 8, 20. PAS was performed to evaluate the tubular injury and the tubular damage score was based on a semiquantitative scale of 0-5+ according to the percentage of tubular damage that reflected tubular necrosis, tubular dilation, cast formation, and loss of the brush border as follows: 0, no lesion; 1+, ≤10%; 2+, 11%-25%; 3+, 26%-45%; 4+, 46%0-75%; and 5+, ≥76%.

### Immunohistochemical analysis

Paraffin sections were evaluated by Immunohistochemistry using a microwave-based antigen retrieval method 21. The primary antibodies used in this study included antibodies against TNF-α, IL-1β, TGF-β1, fibronectin (Santa Cruz Biotechnology, Santa Cruz, CA), F4/80 (AbD Serotec, Kidlington, UK), phospho-Smad3 (Rockland, Immuno-chemicals, Gilbertsville, PA), α-SMA (Sigma, St. Louis, MO), collagen I (Southern Biotech, Birmingham, AL), and phospho-NF-κB/p65 (Abcam, Cambridge, MA, USA). Positive signals were analysed with the quantitative Image Analysis System (Image-Pro Plus 7.0, Media Cybernetics, Bethesda, MD, USA) as described previously 6, 22.

### Western blot analysis

Protein was extracted from the kidney cortex and cultured cells using RIPA lysis buffer. Western blot analysis was performed as described previously 17. Five percent bovine serum albumin (BSA) was used to block nonspecific binding before membrane incubation with primary antibody overnight at 4°C. Antibodies used in this study included primary antibodies against collagen I (Southern Biotech, Birmingham, AL), fibronectin (Santa Cruz Biotechnology), KIM-1 (R&D Systems, Minneapolis, MN), phospho-NF-κB/p65, NF-κB/p65, phospho-Smad3, and Smad3 (Cell Signalling Technology Inc, Danvers, MA, USA). After full washing, membranes were labeled with LI-COR IRDye 800-conjugated secondary antibodies (Rockland Immuno-chemicals, Gilbertsville, PA) for 1 hour at room temperature in 1% BSA/TBST. Signal detection was performed by the Odyssey Infrared Imaging System (LI-COR Biosciences, Lincoln, NE, USA) and quantitatively analysed by normalizing to β-actin by ImageJ software (National Institutes of Health, Bethesda, MD).

### RNA extraction and quantitative real-time PCR

Total RNA was isolated from the renal cortex and cultured cells using TRIzol reagent (Invitrogen) according to manufacturer's instructions, and real-time PCR was performed using Bio-Rad IQ SYBR Green Supermix with Option 2 (Bio-Rad, Hercules, CA, USA) as previously described 16. Primers used in this study for KIM-1, IL-1β, TNF-α, TGF-β1, α-SMA, collagen I, and fibronectin mRNAs have been described previously 8, 16, 17, 21-23. The housekeeping gene β-actin was used as an internal control. The expression level of each mRNA of interest was normalized to that of β-actin and expressed as mean ± S.E.M.

### Statistical analysis

All data obtained in this study are expressed as mean ± S.E.M. Each experiment was repeated at least three times. Statistical analyses were performed with one-way ANOVA followed by Newman-Keuls comparisons using GraphPad Prism 6.0 (GraphPad Software, San Diego, CA, USA). A P-value <0.05 was considered significant.

## Results

### Deletion of Smad3 protects against kidney histological injury in CRP Tg mice with UUO

**Figure 1:**
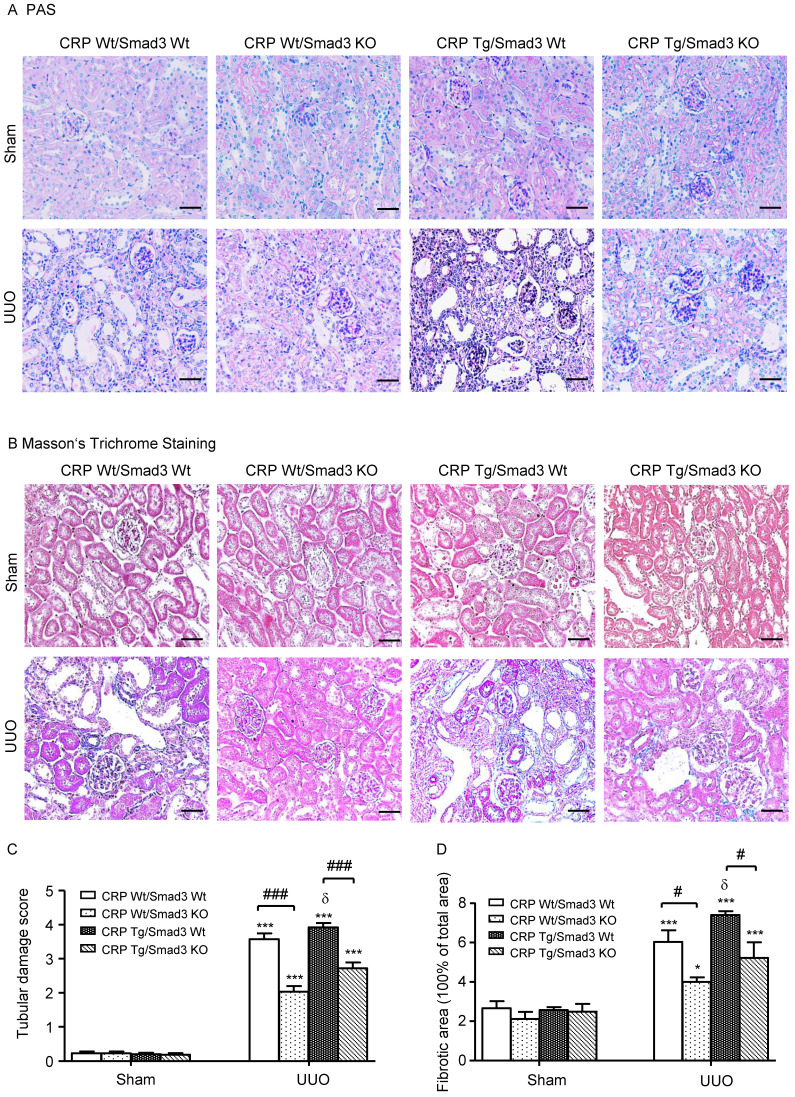
** Renal histopathology of the obstructed kidney after unilateral ureteral obstructive (UUO). (A)** Periodic Acid-Schiff's (PAS) staining and **(B)** Masson's trichrome staining. The semi-quantitative analysis of PAS** (C)** and Masson's trichrome staining **(D)**. Data represent the mean ± SEM for eight mice per group. *p<0.05, ***p<0.001 versus sham group; #*p*<0.05, ###*p*<0.001 as indicated; δ *p*<0.05, versus CRP Wt/Smad3 Wt UUO mice. Scale bars: 50μM. Magnification ×200.

**Figure 2:**
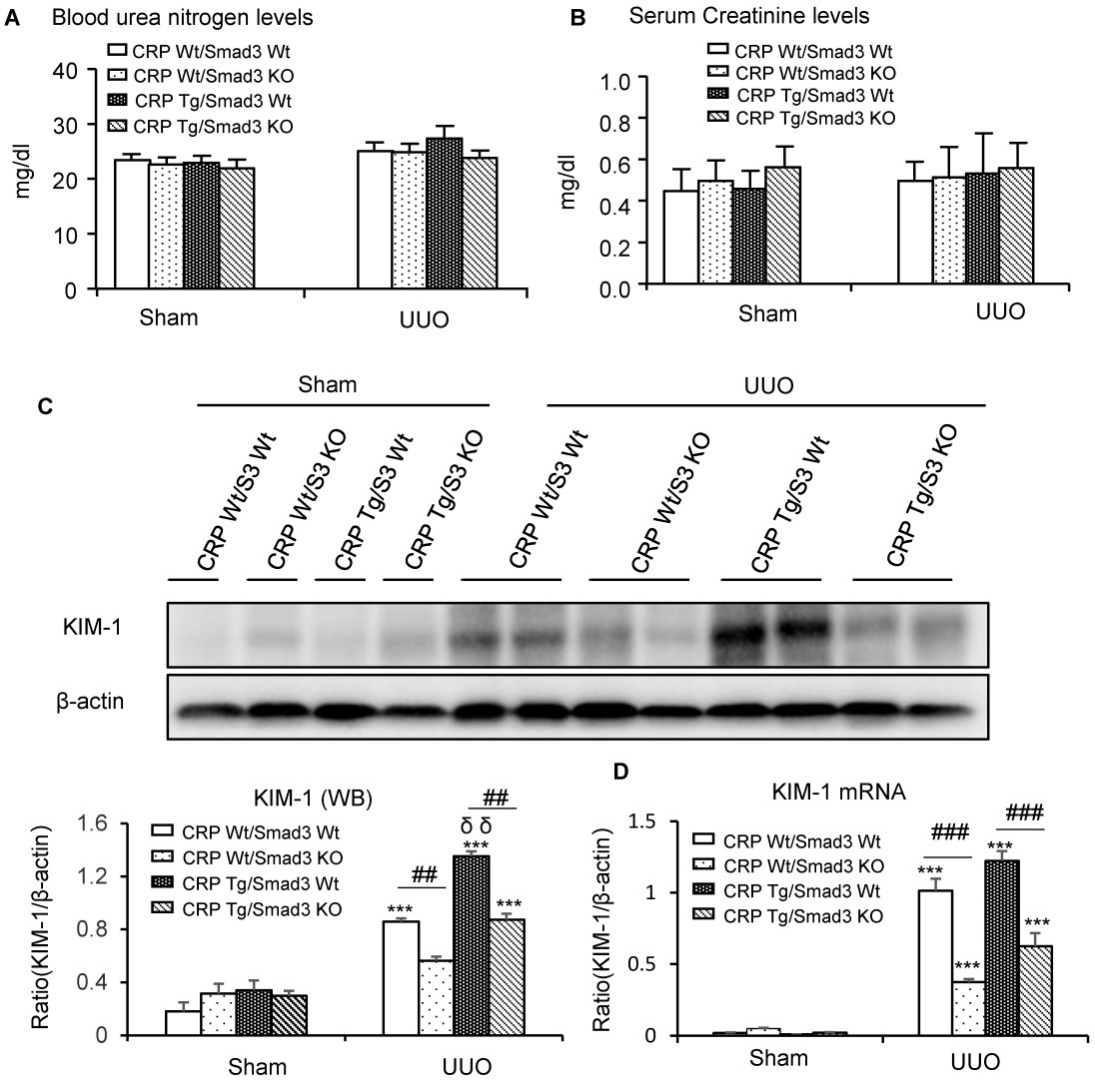
** Kidney injury indexes**. **(A)** BUN and **(B)** Serum creatinine in mice at 7 days of UUO induction. **(C)** KIM-1 protein and **(D)** KIM-1 mRNA in mice at 7 days of UUO induction. Data represent the mean ± SEM for eight mice per group. ***p<0.001 versus sham group; ##*p*<0.01, ###*p*<0.001 as indicated; δδ*p*<0.01 versus CRP Wt/Smad3 Wt UUO mice. BUN: blood urea nitrogen; KIM-1: Kidney Injury Molecule-1; WB: western blot.

Renal histopathology was examined using PAS and Masson's trichrome staining. As shown in **Figure 1**, sham group mice showed normal renal morphology. UUO-induced severe tubulointerstitial damage in Smad3WT/CRP Tg mice. On the contrary, the tubulointerstitial fibrosis index was significantly decreased in Smad3 KO/CRP Tg UUO mice, accordingly (**Figure 1A** and **1B**); These findings were further confirmed by semi-quantification of tubulointerstitial damage observed in PAS and Masson's trichrome-stained sections of kidney tissue (**Figure 1C** and **1D**). We also detected blood urea nitrogen (BUN) and serum creatinine (SCr) levels; results revealed that BUN and SCr were not significantly affected in CRP Tg mice at 7 days after UUO (**Figure 2A** and **2B**). However, the levels of KIM-1 were significantly increased in mouse kidneys at 7 days after UUO, which was further upregulated in CRP Tg/Smad3 WT mice but suppressed in the UUO kidney of CRP Tg/Smad3 KO mice (**Figure 2C** and **2D**). Altogether, these findings indicate that deletion of Smad3 protects against CRP-induced renal histological damage and fibrosis in UUO mice.

### Deletion of Smad3 protects against renal fibrosis and inflammation in CRP Tg mice with UUO

**Figure 3:**
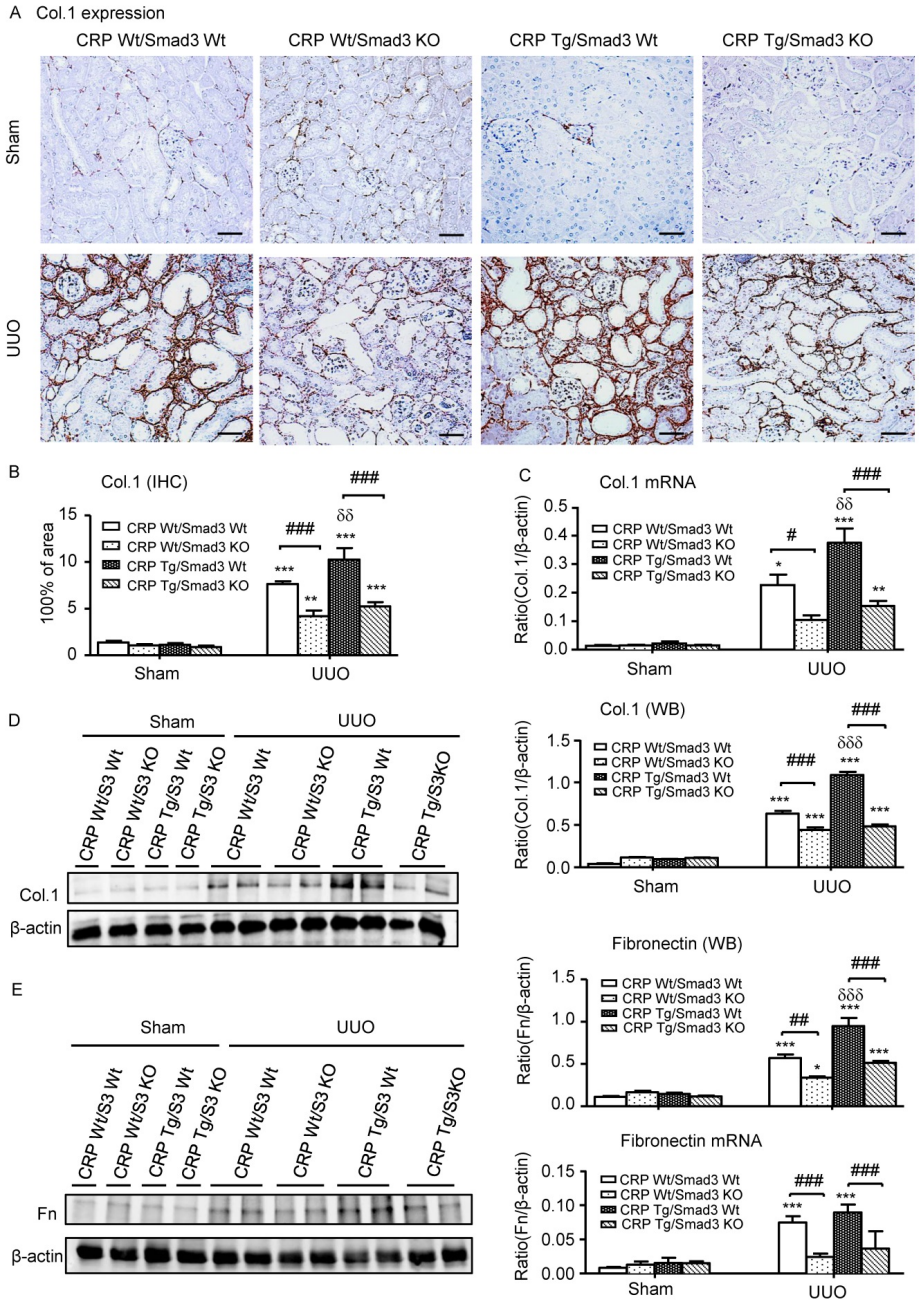
** Deletion of Smad3 protects against renal collagen I and fibronectin expression in CRP Tg mice with UUO. (A)** IHC staining and **(B)** Semi-quantitative analysis of collagen I. **(C)** Quantitative real-time PCR and **(D)** Western blot analysis of collagen I. **(E)** Western blot and quantitative real-time PCR analysis of fibronectin. Data represent the mean ± SEM for eight mice per group. *p<0.05, **p<0.01, ***p<0.001 versus sham group; #*p*<0.05, ##*p*<0.01, ###*p*<0.001 as indicated; δδ*p*<0.01, δδδ*p*<0.001 versus CRP Wt/Smad3 Wt UUO mice. Scale bars: 50μM. Magnification ×200. Col.1: collagen I; Fn: fibronectin; IHC: immunohistochemistry; WB: western blot.

**Figure 4:**
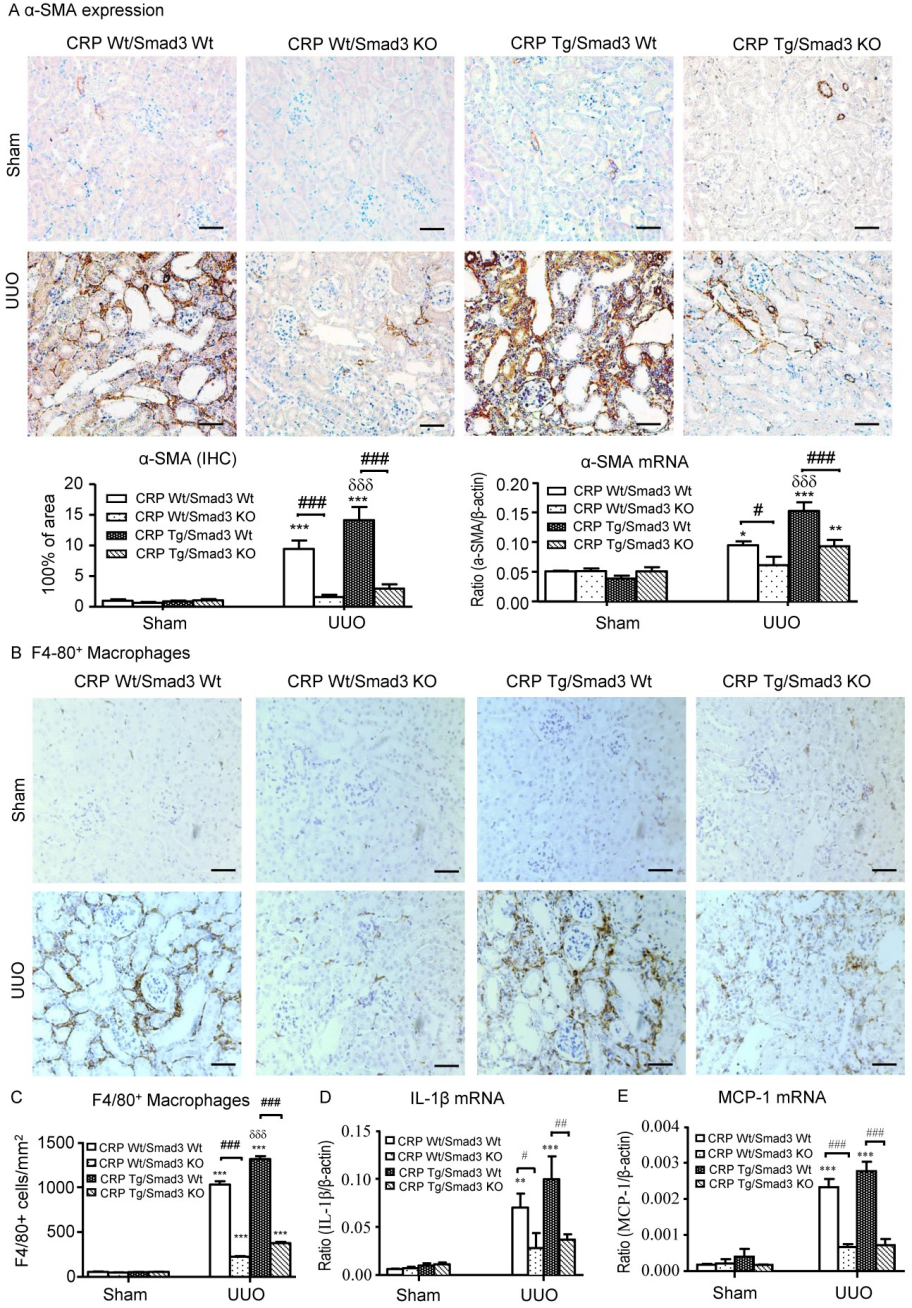
**Deletion of Smad3 protects against α-SMA accumulation and renal inflammation in CRP Tg mice with UUO. (A)** IHC staining and semi-quantitative real-time PCR analysis of α-SMA. **(B)** IHC staining and **(C)** semi-quantitative analysis of F4/80^+^ macrophages. Semi-quantitative real-time PCR analysis of IL-1β **(D)** and MCP-1 **(E)**. Data represent the mean ± SEM for eight mice per group. *p<0.05, **p<0.01, ***p<0.001 versus sham group; #*p*<0.05, ##*p*<0.01, ###*p*<0.001 as indicated; δδδ*p*<0.001 versus CRP Wt/Smad3 Wt UUO mice. Scale bars: 50μM. Magnification ×200. IHC: immunohistochemistry; WB: western blot.

Next, we examined the effect of Smad3 deletion on CRP-induced renal fibrosis and inflammation in UUO mice. Immunohistochemistry, western blotting, and quantitative real-time PCR revealed that, compared with control UUO mice, CRP Tg/Smad3 WT mice developed severe renal fibrosis by increasing both mRNA and protein levels of collagen I, fibronectin, and α-smooth muscle actin (α-SMA) (**Figure 3** and **4A**). In contrast, CRP Tg mice lacking Smad3 (CRP Tg/Smad3 KO) were protected from CRP-exacerbated renal fibrosis by inhibiting expressions of collagen I, fibronectin (**Figure 3**), and α-SMA mRNA and protein (**Figure 4A**).

CRP Tg mice also developed moderate renal inflammation with greater numbers of F4/80^+^ macrophages infiltrating the tubulointerstitial regions in Smad3 WT mice with UUO (**Figure 4B** and **4C**). This was accompanied by a marked upregulation of IL-1β and MCP-1 as shown by real-time PCR (**Figure 4D** and **4E**). In contrast, disruption of Smad3 prevented CRP-exacerbated renal inflammation with sparse F4/80^+^ cell infiltration and attenuated IL-1β and MCP-1 upregulation as seen in CRP Tg/Smad3 KO mice (**Figure 4B-E**).

### Smad3-mediates CRP-exacerbated renal inflammation by enhancing NF-κB signalling in CRP Tg mice with UUO

**Figure 5:**
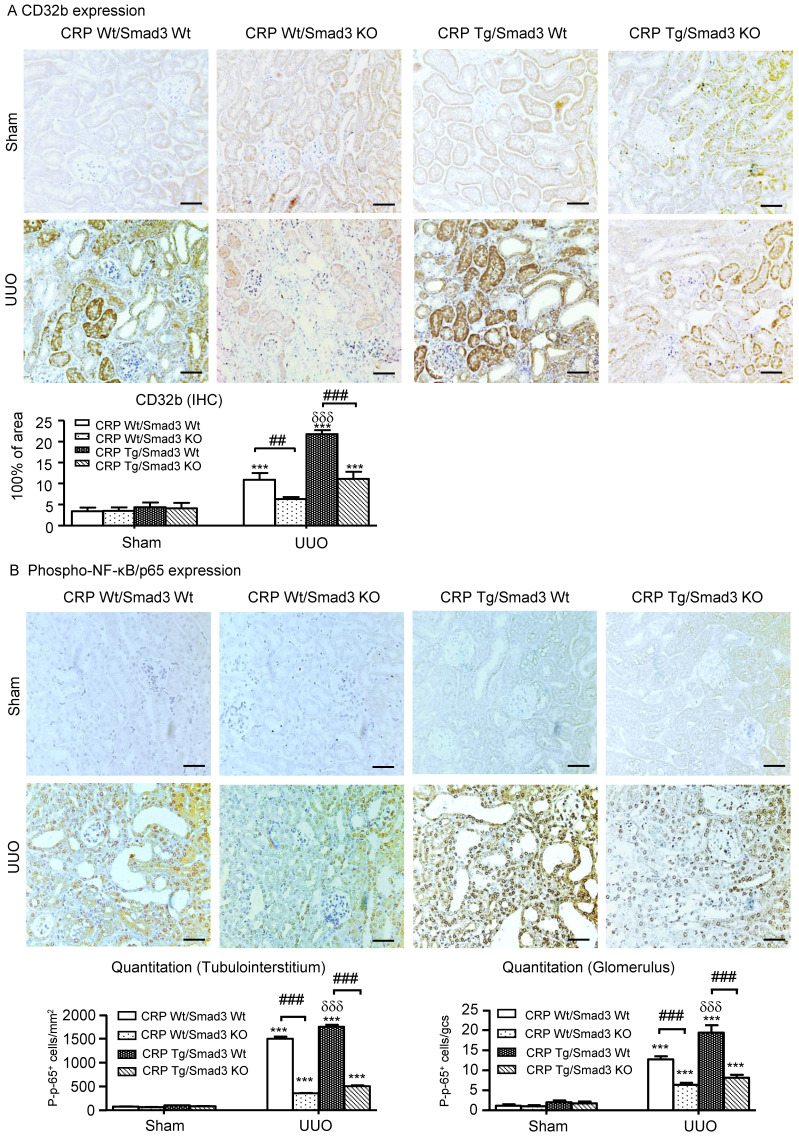
** Deletion of Smad3 attenuates CRP-induced CD32b expression and inhibits NF-κB signalling in the kidney of CRP Tg mice with UUO. (A)** IHC staining and semi-quantitative analysis of CD32b. **(B)** IHC staining and semi-quantitative analysis of nuclear phospho-NF-κB/p65 (p-p65). Data represent the mean ± SEM for eight mice per group. ***p<0.001 versus sham group; ##*p*<0.01, ###*p*<0.001 as indicated; δδδ*p*<0.001 versus CRP Wt/Smad3 Wt UUO mice. Scale bars: 50μM. Magnification ×200. IHC: immunohistochemistry.

We reported CRP can activate NF-κB-mediated renal inflammation and TGF-β/Smad3-mediated renal fibrosis via CD32b in diabetic kidney disease 8. Therefore, we detected the expression of CRP receptor, CD32b in the UUO kidney, immunohistochemistry demonstrated that overexpression of human CRP significantly enhanced renal CD32b expression in CRP Tg/Smad3 WT mice with UUO, which was attenuated in CRP Tg/Smad3 KO mice (**Figure 5A**).

Immunohistochemistry revealed that CRP-exacerbated renal inflammation was associated with activation of NF-κB signalling, as evidenced by a marked increase in levels of NF-κB/p65 in CRP Tg/Smad3 WT UUO mice, but not in CRP Tg/Smad3 KO UUO mice (**Figure 5B**).

### Disruption of Smad3 inhibits CRP-induced renal fibrosis *in vitro*

**Figure 6:**
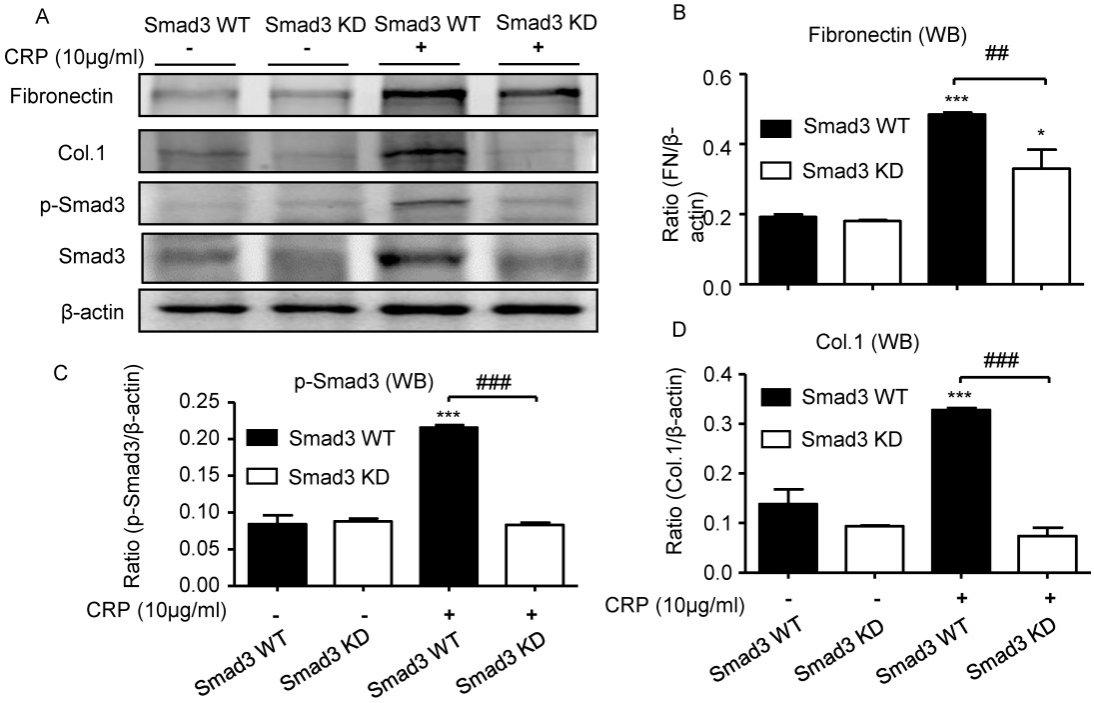
** Knockdown of Smad3 protects against CRP-induced collagen I and fibronectin expression *in vitro*. (A)** Western blot of fibronectin, collagen I, and phospho-Smad3. Semi-quantitative analysis of fibronectin **(B)**, phospho-Smad3 **(C)**, and collagen I **(D)** in both Smad3 WT and Smad3 KD NRK52E cells with or without 10μg/ml CRP stimulation. Data represent the mean ± SEM for at least three independent experiments. *p<0.05, ***p<0.001 compared with untreated Smad3 WT NRK52E cells; ##*p*<0.01, ###*p*<0.001 as indicated. Col.1: collagen I; KD: knockdown; WB: western blot.

We also investigated the functional role of Smad3 in CRP-induced renal fibrosis using Smad3 stable knockdown NRK52E TECs. Results of real-time PCR and western blot analyses revealed that the addition of CRP induced a marked upregulation of collagen I and fibronectin expression in Smad3 WT cells; in contrast, cells lacking Smad3 were protected from CRP-induced fibrotic responses (**Figure 6**).

### Smad3-mediates CRP-exacerbated renal fibrosis via positive feedback of TGF-β/Smad3 signalling pathways *in vivo* and *in vitro*

**Figure 7:**
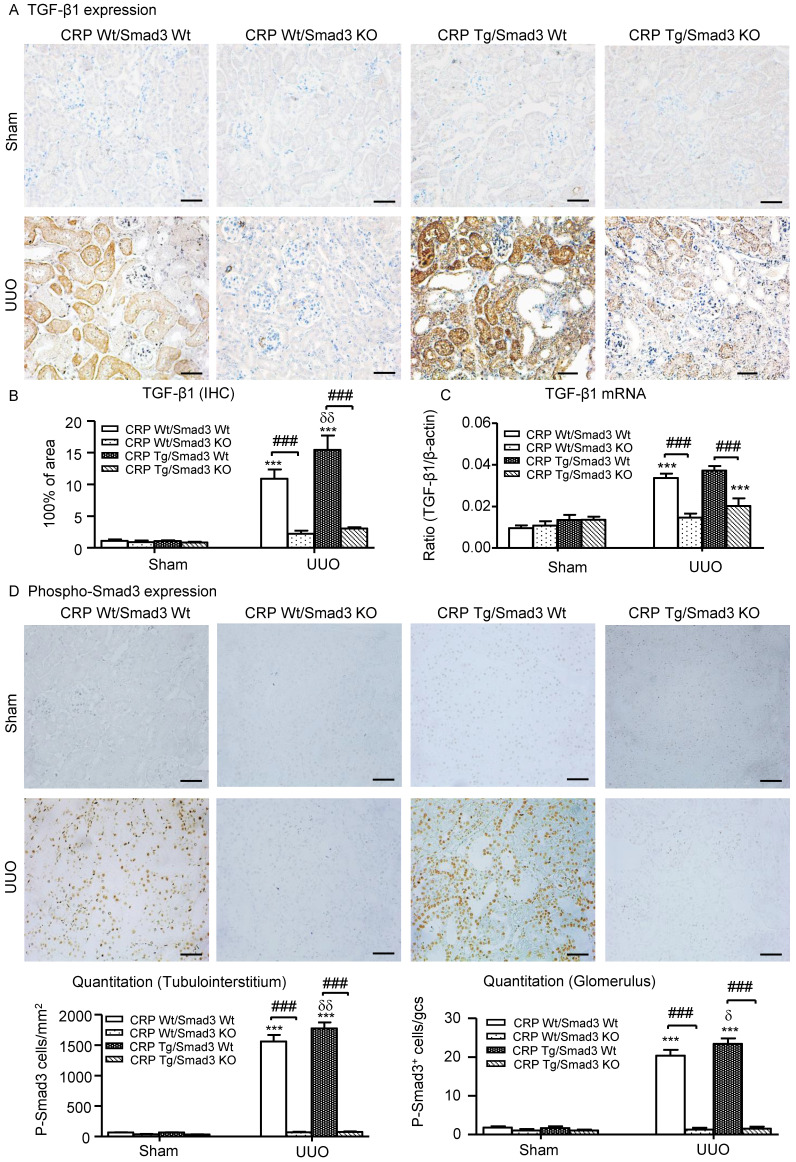
** Deletion of Smad3 prevents CRP-induced TGF-β1/Smad3 signalling in the obstructive kidneys of CRP Tg mice with UUO. (A)** IHC staining and (**B**) Semi-quantitative IHC analysis of TGF-β1. **(C)** Semi-quantitative real-time PCR analysis of TGF-β1. **(D)** Phosphorylation of Smad3 by IHC. Data represent the mean ± SEM for a group of eight mice. ***p<0.001 versus sham group; ###*p*<0.001 as indicated; δ*p*<0.05, δδ*p*<0.01, versus CRP Wt/Smad3 Wt UUO mice. Scale bars: 50μM. Magnification ×200. IHC: immunohistochemistry.

We also explored the mechanisms by which Smad3 KO mice were protected against CRP-induced renal fibrosis *in vivo* and *in vitro*. Since CRP mediates renal fibrosis by stimulating TGF-β1 expression, we hypothesized that CRP-induced TGF-β1 expression may be Smad3 dependent. As illustrated in (**Figure 7A-C**), CRP Tg/Smad3 WT mice markedly upregulated TGF-β1 mRNA and protein, which were found to be associated with strong activation of Smad3 signalling within obstructed kidneys in CRP Tg-Smad3 WT UUO mice (**Figure 7D**). In contrast, deletion of Smad3 resulted in abrogation of CRP-induced upregulation of TGF-β1 and activation of Smad3 in CRP Tg/Smad3 KO mice (**Figure 7**).

## Discussion

In this study, we found that mice lacking Smad3 were protected against CRP-exacerbated kidney injury, including progressive renal fibrosis and inflammation. Results from this study provided evidence for an essential role of Smad3 signalling in the pathogenesis of UUO-induced renal fibrosis and inflammation under high CRP conditions.

An increasing number of studies illustrate that TGF-β1/Smad3 signalling is critical in the progression of renal fibrosis and resultant CKD. We previously reported that Smad3 is a key Smad protein that mediates fibrosis in multiple organs and tissues 3, 17. In addition, inhibition of Smad3 using specific inhibitors (SIS3) can also suppress renal fibrosis 24-27. In the present study, we uncovered that CRP enhances UUO-induced renal inflammation and fibrosis via a Smad3-dependent mechanism. This was supported by the finding that CRP Tg mice lacking Smad3 were protected against CRP-enhanced renal inflammation and fibrosis in a mouse model of UUO. Therefore, results from this study suggest that Smad3 may play an essential role in renal inflammation and fibrosis under high CRP conditions. This may also well explain that high CRP is associated with poor renal outcomes in both experimental and patients with AKI and CKD clinically 6-8, 10. The protection against TGF-β1/Smad3-mediated renal fibrosis and NF-κB-driven renal inflammation are likely mechanisms by which deletion of Smad3 ameliorated kidney injury in CRP Tg mice with UUO.

**Figure 8:**
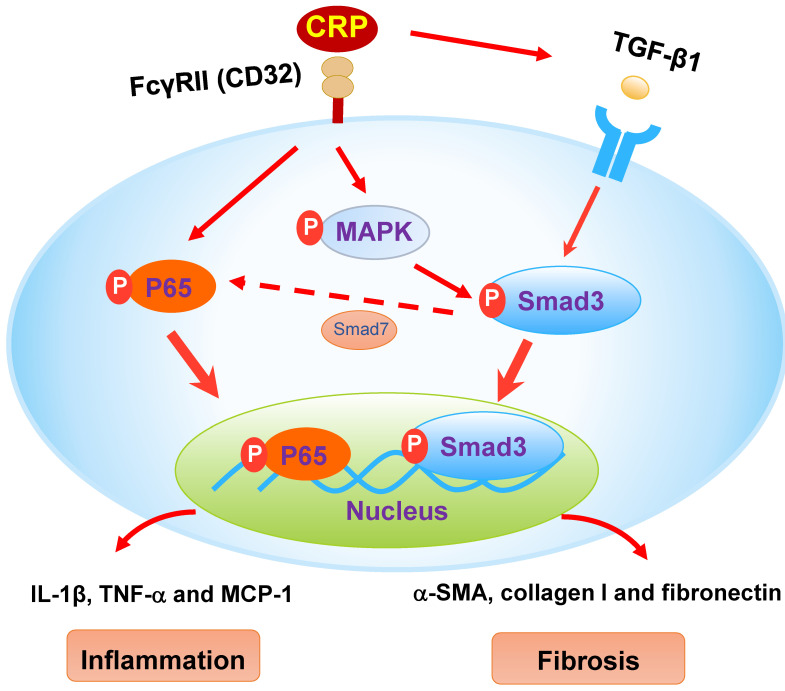
** The schematic diagram illustrates the mechanism.** CRP induces activation of Smad3 and renal fibrosis directly via the CD32-MAPK-crosstalk pathway and indirectly through the TGF-β1-dependent mechanism. CRP induces renal inflammation by CD32-NF-κB dependent mechanism. Simultaneously, the decrease of Smad7 by activation of Smad3 likely enhances renal inflammation via activation of P65/NF-κB.

CRP has been considered as an inflammatory mediator and induces renal inflammation via the CD32-NF-κB-dependent mechanism 6-8. It has been reported that mice overexpressing the human CRP result in severe renal inflammation by markedly up-regulating pro-inflammatory cytokines/chemokines (TNF-α, IL-1β, and MCP-1) and increasing renal infiltration of F4/80^+^ macrophages via an NF-κB-dependent mechanism 6-8. We have previously shown that Smad7 is an inhibitor of NF-κB signaling and is negatively regulated by Smad3 via Smurf2- and Arkadia- dependent ubiquitin degradation mechanism 3, 16, 28, 29. The enhanced Smad7-dependent inhibition of NF-κB signaling may be a mechanism through which CRP Tg mice lacking Smad3 were protected from renal inflammation** (Figure 8)**. In addition, Smad3 is also capable of binding MCP-1 promoter to induce MCP-1 expression 30. Therefore, inhibition of MCP-1 dependent macrophage infiltration may be another mechanism by which Smad3 KO mice were protected from CRP-induced renal inflammation in this study.

We have previously demonstrated that the addition of CPR can induce the early (15 mins) and the late phase (24 hrs) of phosphorylation of Smad3 in CRP treated HK-2 cells 8. Further studies find that CRP-induced the early activation of Smad3 signaling at 15 mins is CD32-Erk1/2 and p38-dependent as it is blocked by a neutralizing antibody to CD32 or the inhibitors of Erk1/2 (PD98059) or p38 (SB203580) but not by a TGF-β1 neutralizing antibody 8. In contrast, CRP-induced the late Smad3 phosphorylation at 24 hrs is TGF-β1-dependent as it can be blocked by a TGF-β1 neutralizing antibody 8. Thus, CRP induces activation of Smad3 and renal fibrosis directly via the CD32b-ERK/p38 MAP kinase-crosstalk pathway and indirectly through the TGF-β1-dependent mechanism 8 **(Figure 8)**. This may also explain why deletion of Smad3 inhibits UUO-induced renal fibrosis in CRP Tg mice.

In conclusion, our findings demonstrate that Smad3 plays an essential role in the pathogenesis of CRP-exacerbated renal injury in UUO-induced obstructive nephropathy. Smad3 mediates renal fibrosis by promoting CRP-induced TGF-β1/Smad3 signalling. Our findings suggest that targeting Smad3 is a promising therapeutic strategy for the treatment of fibrotic kidney disease under high CRP conditions.

## Abbreviations

CRP: C-reactive protein
α-SMA: α-smooth muscle actin
IL-1β: interleukin-1β
NF-κB: nuclear factor κ light chain enhancer of activated B cells
TGF-β1: transforming growth factor-β1
TNF-α: tumour necrosis factor-α
UUO: unilateral ureteral obstruction
